# Advantages and Limitations of Ultrasound as a Screening Test for Ovarian Cancer

**DOI:** 10.3390/diagnostics13122078

**Published:** 2023-06-15

**Authors:** Antonios Koutras, Paraskevas Perros, Ioannis Prokopakis, Thomas Ntounis, Zacharias Fasoulakis, Savia Pittokopitou, Athina A. Samara, Asimina Valsamaki, Athanasios Douligeris, Anastasia Mortaki, Ioakeim Sapantzoglou, Alexandros Katrachouras, Athanasios Pagkalos, Panagiotis Symeonidis, Vasileios-Chrysovalantis Palios, Alexandros Psarris, Marianna Theodora, Panos Antsaklis, George Makrydimas, Athanasios Chionis, Georgios Daskalakis, Emmanuel N. Kontomanolis

**Affiliations:** 1 1st Department of Obstetrics and Gynecology, General Hospital of Athens ‘ALEXANDRA’, National and Kapodistrian University of Athens, Lourou and Vasilissis Sofias Ave, 11528 Athens, Greece; antoniskoy@yahoo.gr (A.K.); paris_per@yahoo.gr (P.P.); ioannisprokopakis@gmail.com (I.P.); thomasntounis@gmail.com (T.N.); hzaxos@gmail.com (Z.F.); savvia_92@hotmail.com (S.P.); thanosdouligeris92@gmail.com (A.D.); anastasiamort@gmail.com (A.M.); kimsap1990@hotmail.com (I.S.); psarris.alexandros@gmail.com (A.P.); panosant@gmail.com (P.A.); gdaskalakis@yahoo.com (G.D.); 2 Department of Embryology, University of Thessaly, Mezourlo, 41110 Larissa, Greece; 3 Department of Internal Medicine, General Hospital of Larisa, Tsakalof 1, 41221 Larisa, Greece; semi_val@hotmail.com; 4 Department of Obstetrics and Gynecology, University General Hospital of Ioannina, University of Ioannina, Stavros Niarchos Str., 45500 Ioannina, Greece; alexkatra1994@gmail.com; 5 Department of Obstetrics and Gynecology, General Hospital of Xanthi, Neapoli, 67100 Xanthi, Greece; sakispagkalos@gmail.com; 6 Department of Obstetrics and Gynecology, Democritus University of Thrace, 6th km Alexandroupolis—Makris, 68100 Alexandroupolis, Greece; simpanthess@gmail.com (P.S.); mek-2@otenet.gr (E.N.K.); 7 Department of Obstetrics and Gynecology, University Hospital of Larissa, Mezourlo, 41110 Larissa, Greece; pampalaios@hotmail.gr; 8 Department of Obstetrics and Gynaecology, University of Ioannina, 45110 Ioannina, Greece; george.makrydimas@gmail.com; 9 Department of Gynecology, Laiko General Hospital of Athens, Agiou Thoma 17, 11527 Athens, Greece; ath.chionis@yahoo.gr

**Keywords:** ovarian cancer (OC), ultrasound, screening test, biomarkers, CA-125

## Abstract

Ovarian cancer (OC) is the seventh most common malignancy diagnosed among women, the eighth leading cause of cancer mortality globally, and the most common cause of death among all gynecological cancers. Even though recent advances in technology have allowed for more accurate radiological and laboratory diagnostic tests, approximately 60% of OC cases are diagnosed at an advanced stage. Given the high mortality rate of advanced stages of OC, early diagnosis remains the main prognostic factor. Our aim is to focus on the sonographic challenges in ovarian cancer screening and to highlight the importance of sonographic evaluation, the crucial role of the operator΄s experience, possible limitations in visibility, emphasizing the importance and the necessity of quality assurance protocols that health workers have to follow and finally increasing the positive predictive value. We also analyzed how ultrasound can be combined with biomarkers (ex. CA-125) so as to increase the sensitivity of early-stage OC detection or, in addition to the gold standard examination, the CT (Computed tomography) scan in OC follow–up. Improvements in the performance and consistency of ultrasound screening could reduce the need for repeated examinations and, mainly, ensure diagnostic accuracy. Finally, we refer to new very promising techniques such as liquid biopsies. Future attempts in order to improve screening should focus on the identification of features that are unique to OC and that are present in early-stage tumors.

## 1. Introduction

Ovarian cancer (OC) is the seventh most common malignancy in women, the eighth greatest cause of cancer-related deaths globally, and the fifth leading cause concerning women in the USA [1,2]. About 19,880 new cases and 12,810 deaths were attributed to OC in the United States in 2022 [2]. On average, 140,000 women worldwide die each year from ovarian cancer [3,4,5]. It also remains the most common cause of death among all gynecological cancers [6]. Even though recent advances in technology have allowed for more accurate radiological and laboratory diagnostic tests, an advanced-stage diagnosis is made in around 60% of all OC cases. Given the high mortality rate of advanced stages of OC, early diagnosis remains the main prognostic factor [2].

As for the ethnicity demographics, the largest occurrence is among Caucasian women (12 per 100,000), followed by Hispanic (10.3 per 100,000), African American (9.4 per 100,000), and Asian women (9.2 per 100,000). In 2018, the prevalence was 6.6 per 100,000 people, and the death rate was 3.9 per 100,000 [7,8]. Notably, OC mortality is much higher among African populations, which may be attributable to socioeconomic determinants of health. As with other diseases, factors such as poverty and inadequate access to health care may influence the outcome of OC [9].

There are as yet no standardized screening tests for OC, and there is a pressing need for novel diagnostic tools, particularly those that can detect the disease at its initial stages while clinical action is still useful. Due to the fact that it is often diagnosed at an advanced stage, recurrence rates are rather high. Despite progress, OC remains the most lethal form of female gynecologic cancer [6]. With an average age of 63 at diagnosis and over 70% of patients presenting with advanced disease, the five-year survival rate is less than 50% [2,10,11,12]. In recent decades there has been a moderate change in the 5-year survival rate, which depends mainly on the disease stage during diagnosis, reaching a percentage of 70–80% in early-diagnosed cases but dramatically decreasing to 20–25% in cases where the disease has been diagnosed at advanced stages [2,12]. Recurrence rates remain high, ranging between 25% and 80% depending on the stage of the disease at diagnosis, despite promising findings from new targeted therapy regimens.

OC can be divided into two subgroups based on its pattern of inheritance. The majority of women with OC have the sporadic variety; however, there is a subset of ovarian cancer that may occur in a familial way. In this particular subset, a substantial family history of ovarian or breast cancer is the most significant risk factor. In general, a hereditary predisposition is responsible at least for 10% of all epithelial OCs, and, more specifically, mutations in the BRCA genes are responsible for 90% of these cases. In these high-risk patients, annual screening with serum CA-125 and transvaginal ultrasound surveillance is indicated. Since the efficacy of these screening approaches is still unclear, prophylactic ovarian surgery is an essential option for patients with confirmed BRCA1 or BRCA2 mutations or a strong family history of breast and/or ovarian cancer. This operation has been shown to lower the likelihood of developing ovarian cancer by 96% and the risk of breast cancer by 53% in individuals who have the BRCA1 or BRCA2 mutation [13].

CA-125 (cancer antigen 125) was established almost forty years ago and has since become the most extensively used and significant biomarker for ovarian cancer. CA-125 is an epitope of MUC16: a 3–5 million Da mucin. On the one hand, its usefulness in OC for the follow-up evaluation of chemotherapeutic effectiveness and prognosis is unquestionable; on the other hand, it is insufficiently trustworthy in early-stage ovarian cancer diagnosis or as a screening tool for the general population [14,15,16,17]. There have been several follow-up strategies proposed; however, according to NCCN guidelines, it has been suggested that follow-up strategies have to be adapted to the tumor’s characteristics and the patient’s needs [18].

The role of ultrasound (U/S) is also well documented in the primary diagnosis of OC and is potentially useful in the detection of OC associated with endometriosis. On the other hand, there is still controversy regarding the use of ultrasound in the follow-up of surgically treated OC. Over the last decade, technological developments have led to a major improvement in U/S imaging quality. The main advantages of U/S are the non-invasive exam procedure, the cost-effectiveness of this technique, which is widely available. Additionally, this technique is a valuable method for monitoring patients with fertility-sparing surgery and a sufficient guide for the biopsy of suspicious lesions in the pelvic area [19].

## 2. Materials and Methods

For this narrative review, authors searched MEDLINE (National Library of Medicine, Bethesda, MD, USA; January 1980 to September 2022) and the Cochrane Register of Controlled Trials (The Cochrane Collaboration, Oxford, UK). An electronic search approach included the phrases ‘Ovarian cancer; Ultrasound; biomarkers; CA-125; screening test’. To find further research that is of interest, references on selected publications and review articles were evaluated. To select possibly relevant papers for this study, the authors evaluated all of the citations returned from the computerized search, incorporating the following exclusion criteria: studies not related to ovarian cancer, non-English language studies, animal studies, and case reports or case series.

## 3. Ultrasound as a Potential Screening Test

One of the most important challenges regarding OC screening is the requirement for effective screening strategies that have a positive predictive value of 10% [20]. In order to achieve this rate of positive predictive values, the screening tool must have a sensitivity of at least 75% and a specificity of 99.6% [21]. Timing is also of great importance in the development of an efficient screening strategy. In this case, OC has no particular time frame for the development of invasive disease nor a particular time frame for the interval stage between stage I and stage III carcinomas [22].

Currently, available biomarkers can be evaluated with samples derived from clinically diagnosed patients and a small number of patients with early-stage or high-grade carcinomas. This is the reason why it is often necessary to make speculations based on cases of advanced disease and not cases of early-stage disease. The difficulty in evaluating the diagnostic ability of screening tests is also evident. The ability of screening tests is correlated to the impact of ovarian cancer mortality rates: a rating that can be confirmed through prospective, randomized, controlled trials. Consequently, very large cohorts are needed in order to evaluate the ability of a certain exam [23].

The ultrasound screening modality allows the detailed imaging of the ovaries as well as the identification of possible morphologic changes that may be recognized as signals for the development of malignancy [24,25,26,27,28]. In order to provide a clear diagnosis, healthcare professionals require certain data, such as the presence of an abnormality in ovarian lesions, the size of the ovaries, blood flow, or the presence of abdominal/pelvic fluid around the ovarian mass, which is evidence that increases the possibility of a tumor being malignant. All of the aforementioned data have been estimated as possible diagnostic factors that could provide the early detection of ovarian cancer. Additionally, any persistent abnormalities that are repeatedly depicted during scanning, between a timeframe of 4 to 6 weeks after the initial screening examination, may reduce the possibility of a false positive result [29,30].

The interpretation of ultrasound images is of great importance since most ovarian masses depicted through an ultrasound examination are benign [26,27]. Consequently, interpretation should be conducted with a strategy that decreases the possibilities of observer variation and thus reduces the frequency of false-positive results. In order to evade these possible pitfalls, a number of screening protocols have been proposed and are utilized in ultrasound examinations. The majority of these protocols are based on morphologic index-based criteria. More specifically, these criteria include findings that can be obtained through a transvaginal ultrasound, such as a cyst wall structure, septation, papillary projections, echogenicity, and ovarian volume, in order to successfully detect the malignancy [31].

### 3.1. Ultrasonographic Assessment of Ovarian Masses

Even though the morphological criteria are similar among the screening protocols, no standardized index is universally accepted, and the systems vary mainly on the type and number of factors that they include. Sassone et al. proposed a widely used index that is based on four different morphological characteristics of an ovarian cyst’s architecture (wall structure, cyst wall thickness, echogenicity, and septae) [31]. (Table 1) A score over nine has high rates of sensitivity and specificity when diagnosing malignancy (100% and 83%, respectively) [32]. Another proposed morphologic index is based on three structural characteristics (septae, ovarian volume, and cyst wall) with lower rates of sensitivity and specificity (89% and 70% correspondingly) [33].

Many clinical trials that focus on the efficiency of ultrasound screening techniques in the diagnosis of OC have been published since the 1980s. These studies have shown that ultrasound is a promising technique; however, a significant variation among the interpretations of the obtained images has been evident [34,35].

#### 3.1.1. IOTA (International Ovarian Tumor Analysis) Model

A very popular used system is the IOTA adnex model, which is based on nine variables. The IOTA (International Ovarian Tumor Analysis) adnex model emerged as a valuable tool for the diagnosis and prediction of ovarian cancer. Several studies have examined the effectiveness of the IOTA model when improving diagnostic accuracy and patient outcomes. The original research by Timmerman et al. (2008) introduced the concept of simple ultrasound-based rules for the diagnosis of ovarian cancer. Their study proposed specific ultrasound criteria, such as the presence of solid areas, bilateral lesions, and ascites, to differentiate between benign and malignant tumors. IOTA models have since evolved from these initial rules, incorporating additional parameters to enhance diagnostic accuracy [24].

**Figure 1:**
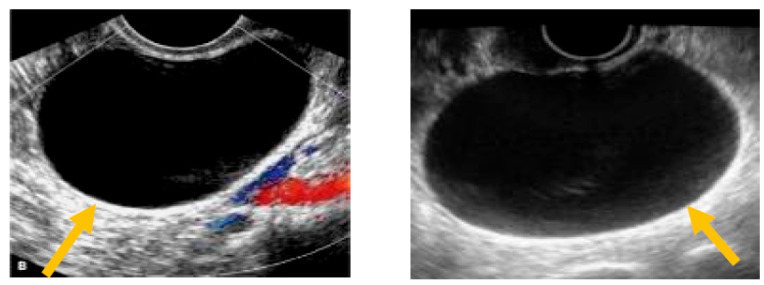
Unilocular cyst. (Yellow arrows indicate the presence of a cyst with one locule, no solid components and no papillary projections, while blood flow is absent).

**Figure 2:**
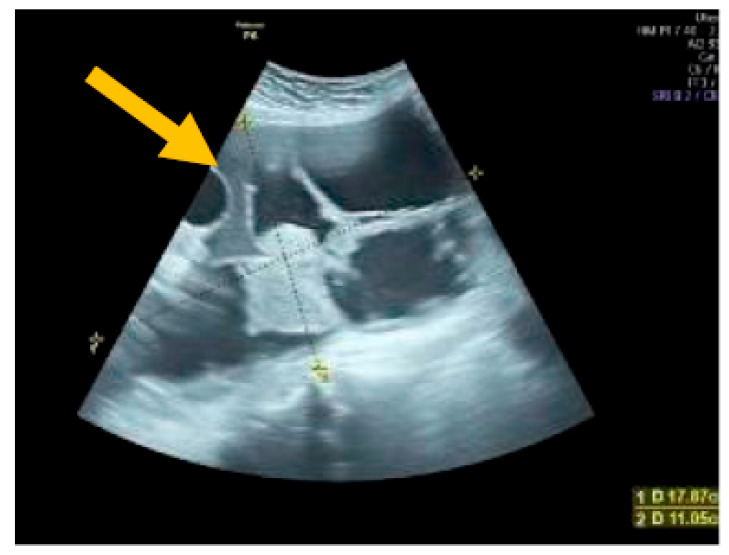
Multilocular solid tumor. (Yellow arrow indicates the presence of a cyst with multiple septums, measurable solid components, and papillary projections.)

**Figure 3:**
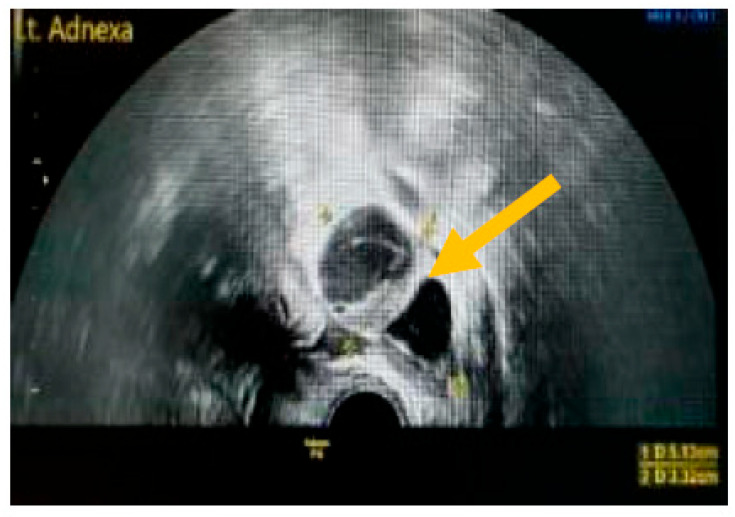
Presence of solid component. (Yellow arrow indicates a multilocular solid cyst with a measurable solid component).

The existence of irregular solid tumors, ascites, at least four papillary structures, an irregular multilocular-solid tumor (diameter at least 10 cm), and very strong blood flow on a color Doppler assessment are malignant indicators, whereas a unilocular cyst, the presence of solid components (max diameter < 7 mm), the presence of an acoustic shadow, a smooth multilocular tumor (max diameter < 10 cm), and the absence of detectable blood flow on the Doppler predispose for the presence of a benign mass [24] (Figure 1, Figure 2 and Figure 3). 

In a study by Timmerman et al. (2010), the IOTA model was validated externally and overtime for its ability to predict ovarian cancer in adnexal masses. These researchers developed logistic regression models based on ultrasound findings and demonstrated that these models effectively discriminated between benign and malignant masses, enhancing the preoperative identification of ovarian cancer. The IOTA models showed good performance in differentiating between early-stage ovarian cancer and benign conditions [25].

The IOTA Simple Rules and SRrisk calculator, developed by the IOTA Group, have been widely utilized in the diagnosis of ovarian cancer. This approach combines different ultrasound parameters to provide a systematic and standardized assessment of adnexal masses. By applying the IOTA Simple Rules, clinicians could classify ovarian masses as either benign, malignant, or inconclusive, enabling more accurate diagnoses and reducing unnecessary surgeries (IOTA Group) [26]. Nunes et al. conducted a meta-analysis in 2014, evaluating the use of the IOTA Simple Rules for diagnosing ovarian cancer. Their findings demonstrated that the IOTA model had high sensitivity and specificity, making it a reliable tool for distinguishing between benign and malignant ovarian tumors [27].

In conclusion, the IOTA simple rules model, consisting of the IOTA Simple Rules has demonstrated its utility in the diagnosis and prediction of ovarian cancer. It offers a standardized approach to evaluating adnexal masses and provides clinicians with valuable information when guiding patient management. By integrating various ultrasound parameters, the IOTA model can enhance the accuracy of ovarian cancer diagnosis, leading to improved patient outcomes and a reduction in unnecessary surgeries. The findings from these studies support the effectiveness and reliability of the IOTA adnex model in clinical practice (Table 2).

#### 3.1.2. RMI (Risk of Malignancy Index)

The Risk of Malignancy Index (RMI) is a widely used scoring system in the assessment of ovarian masses, which aims to estimate the likelihood of malignancy. It incorporates three main components: menopausal status, serum CA-125 levels, and ultrasound findings. Several studies have evaluated the diagnostic performance of RMI when distinguishing between benign and malignant ovarian masses. For example, Jacobs et al. (1990) conducted a study demonstrating the effectiveness of RMI in detecting ovarian cancer. Another study by Tingulstad et al. (1996) validated RMI and found it to be a reliable tool for the risk stratification of adnexal masses. These studies support the value of RMI in clinical practice for decision-making and the management of ovarian masses [36,37].

#### 3.1.3. ROMA (Risk of Ovarian Malignancy Algorithm)

The Risk of Ovarian Malignancy Algorithm (ROMA) is a multivariate algorithm that combines serum CA-125 levels and human epididymis protein 4 (HE4) with menopausal status to predict the risk of ovarian cancer. ROMA has been extensively studied and validated for its diagnostic accuracy in distinguishing between benign and malignant ovarian masses. For example, Moore et al. (2011) conducted a large multicenter study validating ROMA as a reliable tool for the assessment of the risk of ovarian malignancy. Similarly, Karlsen et al. (2011) evaluated ROMA in a prospective study and demonstrated its effectiveness in the preoperative risk stratification of adnexal masses. These studies highlight the utility of ROMA in clinical practice for enhancing the diagnostic evaluation of ovarian masses [38,39].

#### 3.1.4. LR2 (Logistic Regression Model 2)

The Logistic Regression Model 2 (LR2) is a statistical model that incorporates various clinical parameters, including age, menopausal status, ultrasound findings, and serum biomarker levels, to estimate the risk of malignancy in ovarian masses. LR2 has been studied extensively for its diagnostic performance in distinguishing between benign and malignant adnexal masses. For instance, Sayasneh et al. (2011) conducted a prospective multicenter study validating the LR2 model and demonstrating its ability to accurately predict malignancy in ovarian masses. Additionally, Van Calster et al. (2014) performed a systematic review and meta-analysis affirming the robustness and clinical utility of the LR2 model in the preoperative assessment of adnexal masses [40,41].

### 3.2. SRU Consensus for Adnexal Masses

Another valuable tool is the consensus published by the Society of Radiologists in the Ultrasound. Levine et al. (2019) conducted a study to provide updated guidelines for the management of simple adnexal cysts. The authors reviewed the relevant literature and expert opinions to establish consensus recommendations. This study emphasized the importance of appropriate follow-up and reporting practices for these cysts, aiming to improve patient care and reduce unnecessary interventions.

In their update, Levine et al. (2019) highlighted key recommendations for the management of simple adnexal cysts. These included defining the size thresholds for follow-up, establishing appropriate intervals for imaging surveillance, and determining indications for intervention. The authors also provided guidance on reporting terminology and emphasized the need for clear and concise communication among healthcare providers. This consensus update has served as a valuable resource for radiologists and clinicians involved in the evaluation and management of simple adnexal cysts. By standardizing follow-up protocols and reporting practices, healthcare professionals can ensure optimal patient care while minimizing unnecessary interventions and associated risks. The recommendations put forth are based on current evidence and expert consensus, providing a practical framework for the management of simple adnexal cysts.

In summary, the SRU consensus provides updated guidelines for the follow-up and reporting of simple adnexal cysts. These recommendations aim to improve patient care by establishing standardized practices and promoting clear communication among healthcare providers. This study serves as a valuable resource for radiologists and clinicians involved in the management of these cysts, ensuring optimal patient outcomes and minimizing unnecessary interventions [30].

### 3.3. Ultrasound Compared to CT/MRI

There has been a comparison between the diagnostic strategies of ultrasound-based models with CT and MRI in the evaluation of ovarian cancer. A study by Kaijser et al., 2013 [42] provided a comprehensive summary of the International Ovarian Tumor Analysis (IOTA) studies, with a specific focus on comparing the diagnostic strategies of ultrasound-based IOTA models with CT and MRI in the evaluation of ovarian cancer. These studies aimed to improve the diagnostic accuracy and management of adnexal masses.

The findings of the IOTA studies demonstrated that ultrasound-based IOTA models had a comparable or even superior diagnostic performance compared to CT and MRI. Ultrasound, as a widely available and cost-effective imaging modality, has the advantage of real-time visualization and can provide valuable information regarding the morphology, vascularity, and internal characteristics of ovarian tumors. The IOTA models, which utilize specific ultrasound features and scoring systems, showed high sensitivity and specificity when distinguishing between benign and malignant ovarian tumors. The authors highlighted that these models could effectively identify malignancies while reducing unnecessary surgical interventions. Moreover, ultrasound-based IOTA models have the advantage of being non-invasive, allowing for serial examinations and the monitoring of tumor progression over time.

By contrast, CT and MRI are useful adjuncts in certain cases where there is ambiguity or complexity in the ultrasound’s findings. These modalities provide additional information, such as detailed anatomical visualization, an assessment of lymph node involvement, and the evaluation of distant metastases. However, they are generally more expensive, less widely accessible, and may require intravenous contrast administration. The study by Kaijser et al. emphasized that the IOTA models, based on ultrasound findings, can serve as the first-line imaging strategy for evaluating adnexal masses. They offer a practical and efficient approach to the initial assessment of ovarian tumors, enabling accurate diagnosis and appropriate management decisions [42].

In conclusion, IOTA studies, as summarized by Kaijser et al., demonstrate that ultrasound-based IOTA models have comparable or superior diagnostic performance to CT and MRI in the evaluation of ovarian tumors. The IOTA models provide a valuable tool for distinguishing between benign and malignant adnexal masses, leading to improved diagnostic accuracy and appropriate patient management. While CT and MRI have their own strengths and can be useful in certain situations, ultrasound-based IOTA models offer a cost-effective, non-invasive, and widely accessible approach for the evaluation of ovarian cancer [42].

## 4. Current Challenges in the Ultrasound Screening of Ovarian Cancer

It is hypothesized that the biology of ovarian cancer is based on two basic disease subgroups [7,8]. Thus, annual screening with ultrasound can be more beneficial to idle type-I tumors in the early stages [21]. Furthermore, a significant percentage of high-grade serous ovarian cancers was believed to have developed in the fallopian tubes, and more specifically in the fimbriae. These cancers developed initially as very small tumors before metastasizing and, hence, progressing to an advanced stage. Despite knowing the origin of this type of ovarian cancer, the absence of imaging tools that could diagnose it at an early stage is still present [43,44].

### 4.1. False Negatives

Recent calculations with computer models calculated that the median diameter of serous ovarian cancer in the early stages was <3 mm, especially in women that are BRCA-positive. This size is maintained throughout the 4.3-year timeframe when the tumors are estimated to remain at an early stage. However, it is believed that the tumor’s size increases approximately to 9 mm in the late stage of the 4.3 years and, thus, a window of opportunity is provided for the early detection of OC with the help of ultrasound examination. During this window of opportunity, early-stage type-II cancers grow to a measurable size but are still confined to a specific area [45].

Another challenge noted in the literature is that primary tumors with a diameter < 10 mm that has metastasized are difficult to diagnose through data collected by salpingoophorectomies in high-risk patients that are BRCA1/2 positive and have multiple late-stage cancer. These data show that some tumors have metastasizing capabilities before reaching a size that can be detected by ultrasound [46,47].

Ultrasound often fails in the detection of tumors in patients with primal peritoneal cancer, as they have ovaries with a normal appearance and normal volume. Primal peritoneal cancer forms on the ovarian surface but does not cause an increase in the ovarian volume. There are several studies with a sample of women with high-grade serous OC that have minimal or no sonographic malformations after an ultrasound examination. This poses a paradox, especially in patients that are in an advanced stage of the disease [48,49].

### 4.2. False Positives

Another important limitation of sonographic modalities is that sonographic features of early malignant and benign lesions sometimes overlap. The low positive predictive value of ultrasound screening represents a high rate of false-positive results during the assessment of adnexal masses. This is also evident when estimating the positive predictive value of ultrasound as a screening test in the United Kingdom Collaborative Trial of Ovarian Cancer (UKCTOCS) (5% and 235 correspondingly) [50]. However, type I ovarian tumors have a significantly lower mortality rate in comparison to type II tumors. Thus, an increase in the detection of these cases would not have a significant impact on mortality. Consequently, even though early-stage, clinically inactive lesions can be detected through ultrasound with a chance of overdiagnosis, these false positive results would not cause an increase in mortality ratios [51].

### 4.3. Dependence on Operator Experience

A well-reported limitation of the ultrasound examination is the dependence on the operator’s experience. Even though the latest technological advancements have made the examination process significantly easier to perform, there is still significant interobserver variation. This variation may be the result of insufficient experience or a misinterpretation of the physiological anatomy in the ovarian area.

Specifically, a study by Timmerman et al. analyzed the subjective evaluation of ultrasonographic imaging for distinguishing malignant from benign adnexal tumors in order to analyze the interobserver’s variability and to assess the influence of experience on the subjective evaluation of adnexal masses using ultrasonography. For this reason, six observers with various degrees of experience (two experienced, two moderately experienced, and two inexperienced) were called to evaluate 100 static B-mode transvaginal ultrasound pictures of adnexal masses. Each observer was instructed to categorize the masses as benign or malignant and to deliver a diagnostic evaluation. The researchers compared their estimations to the histology diagnoses, which were the gold standard [52].

The results revealed that experienced observers had greater sensitivity (81% and 87%) and specificity (90% and 92%) than moderately experienced (67% and 70% sensitivity; 82% and 87% specificity) and novice observers (57% and 60% sensitivity; 79% and 80% specificity). Positive likelihood ratios were greater for expert observers (8.1 and 10.9) than for moderately experienced or novice observers (3.7 and 5.4). (2.9 and 3.0). In addition, highly experienced observers produced a more precise differential diagnosis. The study concluded by underlying the significance of experience in the subjective evaluation of adnexal masses when utilizing US. Experienced observers provided greater diagnostic precision, highlighting the necessity for ongoing training and education for clinicians adopting this imaging approach [52]. Sonographers acquired the necessary experience after performing a large number of examinations [20].

### 4.4. Limitations in Ultrasound Visualization

There are reported cases where a small percentage of women require a repeat examination after an unsatisfactory visualization in one or both ovaries [53]. A repeat examination may also be required in cases when the view of the iliac vessels is unsatisfactory. Visualization difficulties may be caused by increasing age, previous gynecological surgery (tubal ligation, hysterectomy), obesity, small atrophic ovaries, and normal anatomical variations such as cases where the ovaries are located beyond the range of the instrument [54].

Data collected from 1187 postmenopausal women who underwent transvaginal ultrasound exams revealed that 17.2% of women had inadequate ultrasound imaging of their ovaries, meaning either one or both of their ovaries were not visible. Emphasis must be given to the significance of enhancing ultrasonography techniques in postmenopausal women in order to improve the identification of ovarian cancer. Although inadequate imaging of the ovaries could lead to missed diagnoses, these findings also have significant ramifications for the early diagnosis of ovarian cancer. Additionally, the necessity for healthcare providers to be aware of the factors that can alter the visibility of the ovaries during ultrasound examinations in order to improve diagnostic accuracy and reduce the risk of missed diagnoses is underlined [54]. Depicting fallopian tubes by using ultrasound techniques is challenging, a fact that consists of a very significant restriction, as many high-grade serous ovarian tumors are likely to initiate from the epithelial cells on the fimbriae of the fallopian tubes [21,55].

## 5. Possible Future Improvements

### 5.1. Importance of Quality Assurance Protocols and Guidelines

Enhancing the efficacy and consistency of ultrasonic screening is a crucial element that can reduce the need for repeated examinations and ensure diagnostic accuracy. One way to improve screening is by developing and implementing protocols that can ensure the quality of the procedure. Quality assurance protocols have the potential to lead to higher visualization rates of the ovaries [54,55]. Additionally, medical professionals should follow published guidelines and standards such as those published by the Society of Radiologists in the Ultrasound [30]. These guidelines may help healthcare professionals recognize ovarian cysts in postmenopausal women. A substantial number of these cysts often exceed 3–4 cm and require annual or even biannual inspection. Furthermore, an introduction of strict criteria should be made regarding complex masses. The most important factor relating to complex masses is increased blood flow [24].

### 5.2. Doppler Ultrasound and Transvaginal Color Doppler Imaging

Moreover, the augmentation of existing US imaging techniques to maximize their accuracy, as well as the introduction of new imaging methods that can precisely identify malignant ovarian tumors, constitute two of the most important subjects in imaging research. Doppler techniques and microbubble enhancement have been suggested as methods for enhancing conventional transvaginal U/S.

Under a Doppler ultrasound, early-stage ovarian cancer displays aberrant central ovarian vascularity, which is distinguishable from the typical hilar or peripheral blood flow [56]. The research found that internal vascularity, low pulsatility indices, and low resistive indices, as measured by a Doppler ultrasound, were linked with ovarian cancer [57,58]. Utilizing a risk score based on sonographic observations, recent research has demonstrated that Doppler-based imaging has an 89% sensitivity and 57% specificity for diagnosing invasive and borderline tumors [57]. The efficacy of transvaginal color Doppler imaging (TVCDI) in detecting ovarian cancer was investigated by a study named “Transvaginal Color Doppler Imaging in the Identification of Ovarian Cancer in a Large Study Population”. In this study, 3845 women with adnexal masses had a preoperative TVCDI evaluation. Using a comparison of preoperative and postoperative histopathological diagnoses, the researchers sought to establish the diagnostic performance of TVCDI in identifying malignant ovarian tumors. For detecting malignant ovarian lesions, TVCDI attained a sensitivity of 91.1%, with a specificity of 88.1%, a positive predictive value of 45.7%, and a negative predictive value of 98.9%. Thus, transvaginal color Doppler imaging is a useful diagnostic tool for the detection of ovarian cancer with a diagnostic accuracy that is superior to grayscale sonography, and its high negative predictive value renders it especially successful at ruling out malignancy when the results are negative [59]. 

### 5.3. Microbubble Contrast-Enhanced Ultrasound

Another technique that may improve the detection rate of OC is the utilization of a microbubble contrast-enhanced ultrasound. Contrast microbubbles are micron-sized, intravascular contrast agents composed of a gaseous core surrounded by a solid shell. By functionalizing the shell with binding ligands to specific molecules, a microbubble-enhanced ultrasound can depict molecules such as the kinase insert domain receptor (KDR): one of the vital regulators of neoangiogenesis, which is differentially expressed in several cancers, including breast and ovarian cancer. The development of molecularly targeted contrast microbubbles that can bind to certain molecules produced in cancer has transformed ultrasound into a molecular imaging modality that can enable the enhanced detection, characterization, and monitoring of cancer in preclinical research. This technique may improve early-stage disease detection as it improves ultrasound specificity. On the other hand, patients with tumors originating from the fallopian tubes do not benefit from the microbubble technique [60].

### 5.4. Transvaginal U/S in Combination with Photoacoustic Imaging (PAI)

Transvaginal U/S could also be combined with photoacoustic imaging (PAI) to increase diagnostic precision. PAI’s primary advantage is its ability to capture functional and molecular information in real time from tissues without using radiation or an exogenous contrast [61]. PAI enables the high-resolution identification of angiogenesis and could, therefore, be utilized to diagnose neovascularization in early-stage OC [62,63]. Nevertheless, contemporary PAI methods can only penetrate tissues that are around 5 cm deep, and spatial resolution decreases with increasing depth. Thus, a transvaginal U/S co-registration is necessary for PAI to produce more precise structural data.

### 5.5. Comparison of Ultrasonographic Screening and Multimodal Screening

According to the UK Collaborative Trial of Ovarian Cancer Screening (UKCTOCS), the concurrent use of CA125 combined with the use of US was evaluated. This study examined 202,638 postmenopausal women aged 50 to 74, divided into three groups: no screening, MMS (multimodal screening), and USS (ultrasonic screening). An MMS patient first had a serum CA125 test and then underwent a transvaginal ultrasound to check for ovarian cancer. Contrary, women in the USS group had only transvaginal ultrasounds performed on them. The purpose of this study was to compare the performance of MMS and USS when detecting ovarian cancer and to ascertain the distribution of identified tumors by stage. When comparing MMS with USS for the detection of ovarian cancer, the results showed that the former had a sensitivity of 89.5% and a specificity of 99.8%, while the latter achieved a sensitivity of 75.0% and a specificity of 98.2%. MMS had a 35.1% positive predictive value, but USS was just 2.8% accurate. There is a promise for the early diagnosis of ovarian cancer with these screening approaches, as 47.9% of tumors in the MMS group and 43.3% in the USS group were discovered at stages I or II. The authors concluded that combined blood CA125 testing and a transvaginal ultrasound (MMS) had greater sensitivity and specificity than USS alone in diagnosing ovarian cancer. However, the effect of these screening approaches on ovarian cancer mortality needs more investigation [53].

## 6. Summary

Unnecessary operative procedures performed due to abnormal screening for OC have decreased recently as a result of the standardized protocols introduced in clinical practice [52,53,54,55]. These protocols utilize a combination of ultrasound findings, biomarkers such as CA-125, and a subjective clinical assessment [64,65]. Hence, the false positive rate of the screening modalities has diminished. Despite the evident progress in OC screening, there is room for further improvement, while the false negative rate in ovarian cancer screening remains significant. Ovarian cancer may originate from epithelial cells located on the fimbriae of the fallopian tubes: an anatomical structure that cannot be easily depicted through imaging modalities. This may explain the false negative results of the sonographic assessment [21,60]. Furthermore, due to the fact that only a fraction of tumors that have already metastasized reach a detectable size, ultrasound examination may fail to detect a significant percentage of early-stage ovarian cancers [46,47].

A standardized methodology utilized by physicians may verify that they are constantly examining the same criteria, hence reducing variability and improving the accuracy of their assessments. Strictly standardized models need to be established in clinical practice (such as the IOTA Adnex), while emphasis needs to be given in ultrasound training to the examiners. Moreover, when physicians receive enough training, increasing detection rates of ovarian carcinomas, as well as the precision of ultrasound-based assessments, need to be monitored. Traditional (CA-125) and novel features (Doppler, microbubble contrast-enhanced ultrasound, PAI) can be combined with ultrasound findings and subjective clinical examinations in an effort to improve the early detection of ovarian carcinomas. When the aforementioned characteristics are combined with ultrasound findings and subjective clinical assessments, the capacity to detect ovarian cancer in its earliest stage by doctors could be enhanced. However, it is crucial to highlight that these qualities may not be effective in all circumstances, and their application should be guided by the patient’s and tumor’s unique characteristics.

Overall, the need for a multidisciplinary approach to ovarian cancer detection that combines standardized models, proper ultrasound training, and a variety of diagnostic features is needed so as to improve the accuracy and efficiency of ovarian cancer screening, contributing to improved patient outcomes by guiding clinical decision-making and, ultimately, reducing the mortality rate associated with this disease. Future attempts should focus on the identification of features that are unique to early-stage OC, such as the improvement of imaging tubal lesions, as well as the clinical implementation of liquid biopsies to assess the circulating tumor DNA and the circulating tumor cells: a potentially useful tool in the screening and diagnosis of OC.

## Figures and Tables

**Table 1:** Sassone scale for morphologic ovarian characteristics.

Value	Inner Wall Structure	Wall Thickness	Echogenicity	Septa
1	Smooth	Thin ≤ 3 mm	Sonolucent	None
2	Irregularities ≤ 3 mm	Thick > 3 mm	Low echogenicity	Thin ≤ 3 mm
3	Papillaries > 3 mm	Not applicable, mostly solid	Low echogenicity with echogenic core	Thick > 3 mm
4	Not applicable, mostly solid	-	Mixed echogenicity	-
5	-	-	High echogenicity	-

**Table 2:** IOTA simple rules.

Malignant-rules	Irregular solid tumor Presence of ascites ≥4 papillary structures Irregular molecular solid tumor with max diameter ≥ 10 cm Very strong blood flow
Benign-Rules	Unilocular cyst Presence of solid component with max diameter < 7 mm Presence of acoustic shadows Smooth molecular tumor with max diameter < 10 cm No blood flow

## Data Availability

No new data were created or analyzed in this study. Data sharing is not applicable to this article.

## Abbreviations

U/S	Ultrasound
CT	Computed Tomography
OC	Ovarian Cancer
BRCA	Breast Cancer gene
NCCN	National Comprehensive Cancer Network
IOTA	International Ovarian Tumor Analysis
UKCTOCS	United Kingdom Collaborative Trail of Ovarian Cancer
CA-125	Cancer Antigen 125
MUC16	Mucin 16
KDR	Kinase insert Domain Receptor
PAI	Photoacoustic Imaging
USS	Ultrasonic Screening
MMS	Multimodal Screening
TVCDI	Transvaginal Color Doppler Imaging
